# *Plasmodium falciparum* genetic variation of *var2csa* in the Democratic Republic of the Congo

**DOI:** 10.1186/s12936-018-2193-9

**Published:** 2018-01-24

**Authors:** Robert Verity, Nicholas J. Hathaway, Andreea Waltmann, Stephanie M. Doctor, Oliver J. Watson, Jaymin C. Patel, Kashamuka Mwandagalirwa, Antoinette K. Tshefu, Jeffrey A. Bailey, Azra C. Ghani, Jonathan J. Juliano, Steven R. Meshnick

**Affiliations:** 1 Medical Research Council Centre for Outbreak Analysis & Modelling, Department of Infectious Disease Epidemiology, Imperial College London, London, UK; 2 Program in Bioinformatics and Integrative Biology, University of Massachusetts, Worcester, MA USA; 3 Division of Transfusion Medicine, Department of Medicine, University of Massachusetts, Worcester, MA USA; 4 Institute for Global Health and Infectious Diseases, School of Medicine, University of North Carolina at Chapel Hill, Chapel Hill, USA; 5 Department of Epidemiology, Gillings School of Global Public Health, University of North Carolina at Chapel Hill, Chapel Hill, USA; 6 Kinshasa School of Public Health, Hôpital General Provincial de Reference de Kinshasa, Kinshasa, Democratic Republic of Congo; 7 Community Health, Kinshasa School of Public Health, School of Medicine, University of Kinshasa, Kinshasa, Democratic Republic of Congo; 8 Division of Infectious Diseases, University of North Carolina at Chapel Hill, 130 Mason Farm Road, Chapel Hill, 27599 USA; 9 Curriculum in Genetics and Microbiology, University of North Carolina at Chapel Hill, 321 South Columbia Street, Chapel Hill, NC 27516 USA

## Abstract

**Background:**

The Democratic Republic of the Congo (DRC) bears a high burden of malaria, which is exacerbated in pregnant women. The VAR2CSA protein plays a crucial role in pregnancy-associated malaria (PAM), and hence quantifying diversity at the *var2csa* locus in the DRC is important in understanding the basic epidemiology of PAM, and in developing a robust vaccine against PAM.

**Methods:**

Samples were taken from the 2013–14 Demographic and Health Survey conducted in the DRC, focusing on children under 5 years of age. A short subregion of the *var2csa* gene was sequenced in 115 spatial clusters, giving country-wide estimates of sequence polymorphism and spatial population structure.

**Results:**

Results indicate that *var2csa* is highly polymorphic, and that diversity is being maintained through balancing selection, however, there is no clear signal of phylogenetic or geographic structure to this diversity. Linear modelling demonstrates that the number of *var2csa* variants in a cluster correlates directly with cluster prevalence, but not with other epidemiological factors such as urbanicity.

**Conclusions:**

Results suggest that the DRC fits within the global pattern of high *var2csa* diversity and little genetic differentiation between regions. A broad multivalent VAR2CSA vaccine candidate could benefit from targeting stable regions and common variants to address the substantial genetic diversity.

**Electronic supplementary material:**

The online version of this article (10.1186/s12936-018-2193-9) contains supplementary material, which is available to authorized users.

## Background

Pregnancy-associated malaria (PAM) is a major public health concern. In areas of stable malaria transmission in sub-Saharan Africa approximately one in four pregnant women have evidence of malaria infection at time of delivery [1, 2]. PAM is detrimental to both mother and child, placing the mother at increased risk of severe anaemia whilst increasing the chance of adverse birth outcomes, including stillbirth, preterm birth and low birth weight (LBW) [1–3].

The adverse effects of PAM are mediated by the sequestration of infected erythrocytes in the placental microvasculature through binding of VAR2CSA—a large and genetically diverse parasite protein expressed during pregnancy-to human chondroitin sulfate A (CSA) [4, 5]. Naturally occurring anti-VAR2CSA antibodies provide partial protection against future episodes of PAM, such that primigravid women are most susceptible and risk of severe infection and LBW decreases in subsequent pregnancies [4, 6, 7]. Vaccines against VAR2CSA are currently undergoing initial trials [8–10].

Extensive effort has gone into characterizing the particular sub-region of the VAR2CSA protein responsible for binding CSA and inducing a protective immune response. These efforts have led to the identification of ID1-DBL2X; a 1.6 kb segment encoding the minimal binding epitope [11]. ID1-DBL2X has been shown to raise antibodies that abrogate the adhesion of infected erythrocytes to CSA with the same efficacy and specificity as the full-length extra-cellular protein, while maintaining high cross-reactivity to multiple parasite lines [12]. Both leading PAM vaccine candidates, PlacMalVac and PRIMALVAC, use recombinant proteins that target overlapping constructs of this region [9, 10].

The ability of any such vaccine to have a sustained impact on malaria will depend on the extent of antigenic variation in the vaccinated population, which in turn depends on the level of sequence polymorphism in the *var2csa* gene. Studies into global diversity at the *var2csa* locus have identified extremely high sequence polymorphism, with evidence that diversity is being maintained by balancing selection [13–16]. Furthermore, this high level of diversity occurs against the backdrop of a major dimorphic split in the N-terminal segment of the VAR2CSA protein, leading to multiple sequence clusters, each of which has been found to associate with a different level of parasitaemia [16] and a different risk of poor birth outcomes [17]. Sequence polymorphism at the *var2csa* locus is, therefore, both functionally relevant in vaccine design and clinically relevant in understanding the basic epidemiology of PAM.

The Democratic Republic of Congo (DRC) bears one of the highest malaria burdens in sub-Saharan Africa, with over 1 million *Plasmodium falciparum* affected pregnancies each year [18]. Transmission is stable throughout the country, and prevalence is estimated at 34.1% on average by PCR [19]. The majority of studies into the genetics of *P. falciparum* in DRC have focussed on issues of drug resistance (see Additional file 1). Studies into *dhps* mutations, which confer resistance to sulfadoxine, have found distinct geographic clustering of the most highly resistant haplotypes in the east of the country [20, 21]. In contrast, countrywide studies into neutral genetic variation [22] and variation in the *pfama1* gene [23] have found little signal of population structure or isolation by distance even over large geographic scales. To date, no study has explored the geographic and genetic structure of *var2csa* in DRC, and so it is unknown what challenges may lie ahead in terms of vaccine design.

This study focused on quantifying genetic variation at the *var2csa* locus in samples obtained from the 2013–14 Demographic and Health Survey (DHS); a large, cross-sectional study separated into spatial clusters spanning the DRC. Central aims of this study were: (1) to explore *var2csa* diversity in these samples in the context of global diversity at this locus, (2) to quantify the level of spatial structure in the DRC, and (3) to determine what epidemiological factors (if any) are predictors of observed levels of diversity at this locus. These questions will be important for any future interventions aimed at reducing PAM in the DRC.

## Methods

### Sample collection

**Fig. 1:**
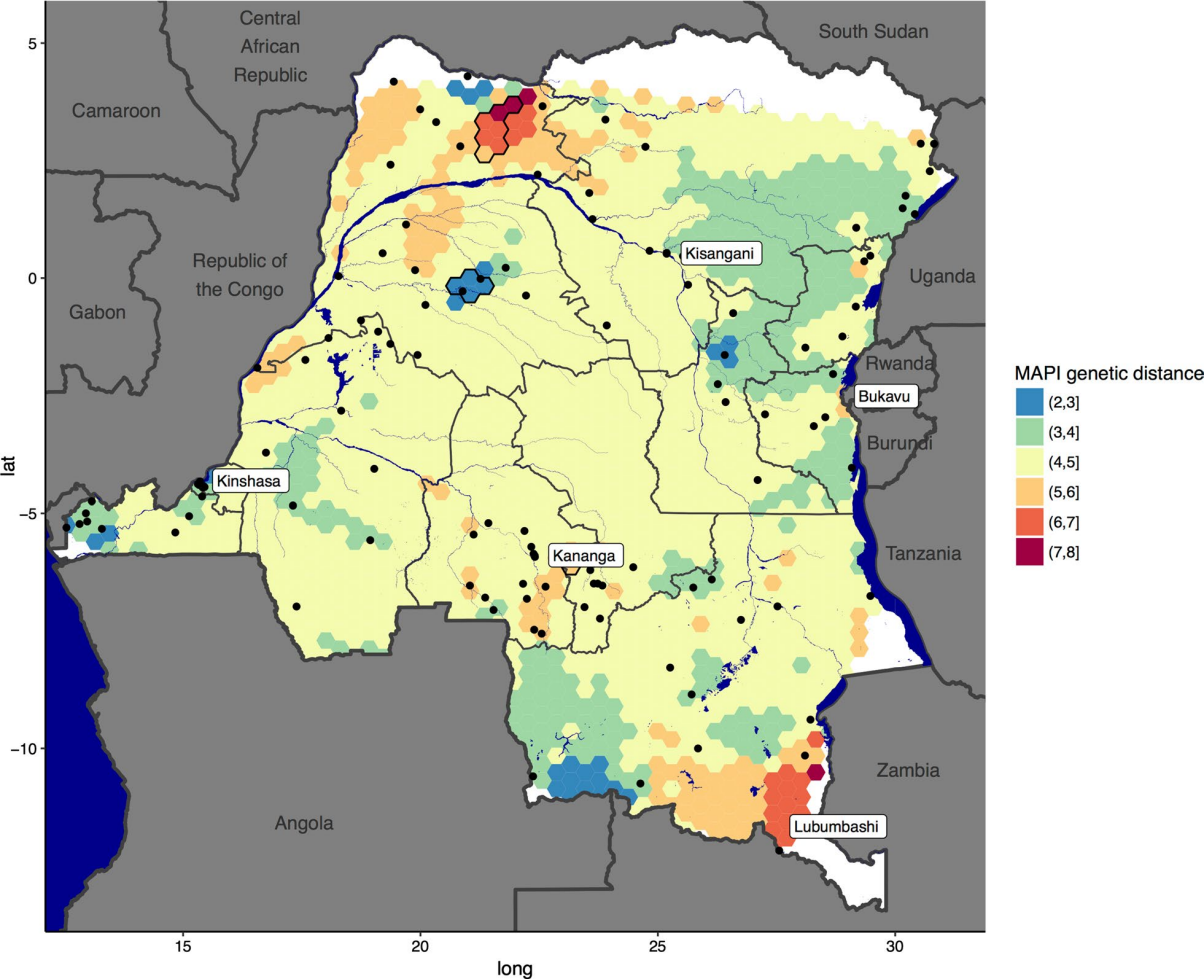
Result of MAPI analysis. Black circles represent the 115 DHS clusters used in the analysis, grey borders give national and sub-national boundaries, and dark blue regions indicate major water bodies. The colour of each cell represents a weighted average of the pairwise genetic distance transecting that cell (white = no data). The two groups of outlined cells in the north have genetic distance that is statistically significant in permutation testing

Samples from children under 5 years of age were collected as part of the 2013–2014 DHS study [19]. Heel- or finger-prick blood from each child was used to prepare smears for identification of malaria parasites via light microscopy, and to prepare dried blood spots (DBS) using previously described methods [24]. DBS were transported to the University of North Carolina, where genomic DNA (gDNA) was extracted using previously described methods [25]. Each gDNA sample was tested in duplicate in a duplex quantitative real-time PCR assay targeting the *P. falciparum* lactate dehydrogenase gene (*pfldh*) [26] and, as a control, the human β-tubulin gene [27]. The full DHS study was conducted in 540 clusters at the neighbourhood or village level, and the current study was performed on a subset of these clusters. A total of 115 clusters were selected that contained at least three parasitaemic children (i.e. smear and *pfldh*-*positive*), and that were spatially representative of the whole country (Fig. 1). For each of the 115 clusters, all the gDNA samples were pooled in equimolar volumes to ensure equal contribution to the pool of each infected child.

### DNA amplification and sequencing

A 400 bp region of the binding domain of VAR2CSA was amplified by primers listed in Table 1. Samples from the 115 clusters were pooled and PCR amplified in technical duplicates. The forward primer included replicate specific molecular identifiers (MIDs) which allowed amplicon pooling prior to library preparation using the NEBNext Ultra DNA Library Prep Kit for Illumina (New England Biolabs, United States). The Illumina primers included Illumina’s dual barcoding system. This approach enabled multiplexing of pooled samples and libraries into one 2 × 300 sequencing run on the Illumina MySeq platform at the University of North Carolina High Throughput Sequencing Facility.

**Table 1:** Primers used

Forward primer	Reverse primer
ATCATGGTGGAACACGAACA	GTACCCGCTTTACGGTTTCG

### Haplotype determination

The paired-end sequences were stitched by FLASH [28], and haplotype determination was performed using the program SeekDeep [29] using a quality cut-off of Q30 > 0.75. In brief, sequence reads were first demultiplexed according to MIDs into amplicon-specific data, resulting in two independent amplicon reads sets for each of the 115 pools, after which MIDs were trimmed and clustered. Only predicted haplotypes that appeared in both replicates for each pool and occurred at a frequency of ≥ 0.5% were utilized to determine the most likely haplotypes within each pool. SeekDeep also removed any haplotypes that may have resulted from chimerization during PCR. This pipeline resulted in the identification of 583 unique haplotypes throughout the 115 clusters.

### Phylogenetic analysis

The similarity between sequences in this study and previously identified *var2csa* variants was established by conducting a BLAST search of all 583 sequences individually against the NCBI database. 81% of top BLAST hits came from just two published collections, which were downloaded and incorporated, bringing an additional 84 sequences from Benin [16] and 36 sequences from Senegal [30, 31]. The corresponding region of the 3D7 genome was also downloaded from PlasmoDB [32], leading to an expanded data set of 704 sequences. These additional sequences were used to improve sequence alignment, and to root DRC sequences relative to wider African variation.

**Fig. 2:**
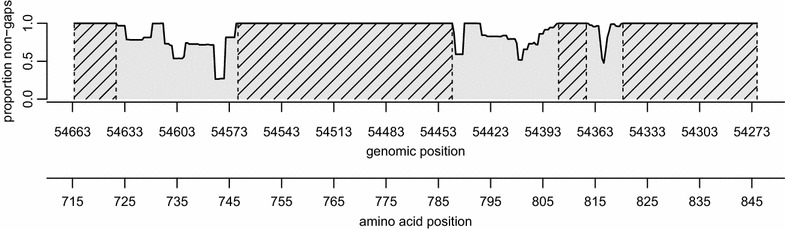
Schematic of *var2csa* sequence variation. The proportion of non-gap sequences at each locus (i.e. one minus the proportion of gaps) is plotted for all nucleotide positions relative to *Pf*3D7 chromosome 12 and corresponding amino-acid positions. The four shaded regions have zero gaps, and were used in all analyses after the sequence alignment step. Our sequences mapped to 3D7 positions 54,271:54,663 and amino acid positions 715:845

Initial attempts at multiple sequence alignment failed due to difficulties in aligning several highly variable regions containing large numbers of insertions and deletions, and so an iterative approach to alignment was used. First, all pairwise alignments were produced using the *msa* package in the R programming language [33, 34]. Sequences were then grouped into 11 groups using agglomerative hierarchical clustering, such that sequences with a high proportion of matched bases clustered together. Multiple alignment was then carried out on each group independently using the ClustalW algorithm in MEGA7 [35], and any obvious alignment errors were removed by hand. Aligned groups were then combined using profile alignment in ClustalX [36]. Regions with large numbers of insertions or deletions were identified by counting the proportion of gaps at each locus, and subsequent analyses were restricted to four contiguous regions with zero gaps to ensure that results were not influenced by ambiguous alignment in a few highly variable regions (Fig. 2).

A neighbour-joining tree was constructed in MEGA7 using default substitution parameters, and bootstrapped with 100 replicates. Focusing on the 583 sequences from DRC hereafter, Tajima’s D statistic was calculated [37] and a Z-test for selection was carried out based on relative rates of synonymous and non-synonymous variation in MEGA7 [38].

### Spatial and epidemiological analysis

Sequence variants were grouped into the 115 DHS clusters from which they derived. The number of clusters occupied by a variant was correlated against the top BLAST hit percentage for that variant, to establish whether variants that were common in DRC also tended to be similar to previously identified sequences. Heterozygosity within and between clusters was calculated, and nearness to fixation was measured via G_ST_ [39]. Isolation by distance was explored by regressing Nei’s genetic distance [40] against geographic distance, and more subtle signals of spatial patterning—for example barriers to gene flow—were explored using the program MAPI [41]. A matrix of pairwise genetic distances between DHS clusters was analysed using MAPI to produce a smoothed surface indicating spatial connectivity between regions, coupled with a permutation test to identify regions of significantly high or low connectivity. MAPI was run using default parameters (*β* = 0.25, eccentricity = 0.975, error circle = 0.1), and statistical significance was assessed using 1000 permutations at a threshold of *α* = 0.05.

Epidemiological analyses focused on finding covariates that explained a significant proportion of the observed genetic variation through generalized linear modelling (GLM). Predictor variables included prevalence of a cluster (defined as the proportion of PCR-positive individuals), sample size, and median cycle threshold (C_t_) value; C_t_ values represent the number of PCR cycles needed before a signal is detected in the DNA amplification stage, and therefore C_t_ is roughly inversely proportional to the amount of nucleic acid in the sample. Results of the DHS questionnaire for each cluster were also downloaded from the DHS website [42], and both “province” and “urbanicity” of the cluster (categorized as “large city”, “small town” and “countryside”) were included as potential predictors. The response variable was the allelic richness, defined as the number of unique haplotypes in a given cluster. A range of GLMs were considered, starting with a full model containing all the predictors listed above and all second-order interaction terms (5 + 25 terms in total). Backwards stepwise selection was then used to identify the model with the lowest Akaike information criterion (AIC). This process was repeated for both Poisson and negative binomial error structures. A separate analysis explored the relationship between C_t_ value and prevalence using both a simple linear model and a mixed-effects model with cluster modelled as a random effect. Mixed effects models do not generate *p* values for individual variables, and so 95% confidence intervals for fitted values were obtained by simulation using the *confint()* function in the R package *lme4* [43].

## Results

### Phylogenetic analysis

Sequences ranged from 365 to 506 bp (median 413), and had an elevated average GC concentration of 36% compared with the genome-wide average of around 19% [44]. BLAST results revealed that 39 of the 583 sequences (7%) were perfect matches to previously identified *var2csa* accessions in the NCBI database, and the remaining 544 sequences had an average similarity of 87% to their top BLAST hit. After combining our sequences with collections from Benin and Senegal downloaded from NCBI, and carrying out multiple sequence alignment as detailed above, three highly variable regions were clearly visible amid four regions with zero gaps (Fig. 2). Subsequent analyses focused exclusively on these zero-gap regions, trading off the additional information contained in the pattern of insertions and deletions between sequences with increased reliability of any detected polymorphisms. The neighbour-joining tree revealed very little phylogenetic signal (see Additional file 2), and corresponding bootstrap values revealed little confidence in the tree, with only 11% of clades having > 80% confidence. Tajima’s D was estimated at 1.95, indicating that the allele frequencies of variants are more evenly distributed than expected (fewer variants at high frequency). The Z-test for selection revealed an excess of synonymous mutations (dN–dS = − 1.445), although this was non-significant at the 5% threshold (*p* = 0.151).

### Spatial and epidemiological analyses

After grouping sequence variants into the 115 DHS clusters from which they originated, there was a median 7 variants per cluster (range 1–28). Variants that were present in many clusters also tended to have high BLAST scores (see Additional file 3), and this correlation was significant (ρ = 0.176, p < 0.001). The most common variant in the DRC was a 100% match to a variant previously identified from Senegal [31]. Heterozygosity was high both within DHS clusters (mean = 0.62, sd = 0.21) and between clusters (mean = 0.81, sd = 0.07), and pairwise G_ST_ was also high (mean = 0.25, sd = 0.13). However, regression of genetic distance against geographic distance revealed no simple signal of isolation by distance (see Additional file 4), and the relationship was non-significant (p = 0.217). Analysis in MAPI also showed relatively uniform connectivity between clusters spanning the DRC, with no major barriers to gene flow and just one small area of significantly high genetic distance (i.e. low connectivity) in the Nord-Ubangi province, and one area of significantly low genetic distance (i.e. high connectivity) in the Tshuapa province (Fig. 1).

**Fig. 3:**
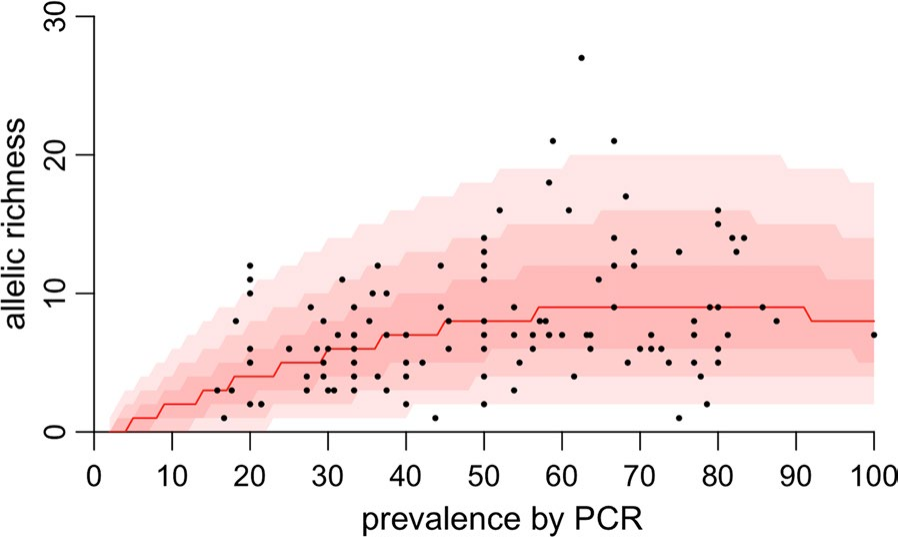
Observed and model-predicted relationship between prevalence and allelic richness. Black circles represent data for each of the 115 clusters. Red shaded regions indicate the 95, 80 and 50% predictive intervals of the best-fitting model, and the red line represents the median prediction. The best fitting model also includes a sample size term, and so these predictions were generated assuming a sample size of 14 (the median sample size in the data)

In the analysis of allelic richness, GLMs with negative binomial error structures consistently yielded better model fits (lower AIC values) compared with the Poisson model, suggesting that allelic richness is over-dispersed within clusters. The best-fitting GLM included just 3 out of a potential 30 predictors, including linear and quadratic terms for prevalence and a linear term for sample size (Table 2). Neither median C_t_ value per cluster nor the demographic parameters “province” and “urbanicity” were identified as important predictors of observed genetic variation. Overall the best-fitting GLM was a good fit to the data (Fig. 3, see also Additional file 5). In the separate analysis of C_t_ values, the mixed effects model gave a better fit (AIC = 4346) than the simple linear model (AIC = 4389), and C_t_ value was found to have a negative relationship with prevalence [coefficient = − 2.603, CI (− 4.359, − 0.846)], suggesting that higher quantities of DNA tend to occur in samples obtained from high prevalence clusters.

**Table 2:** Parameters of best-fitting GLM (Negative-binomial model: AIC = 634.8, compared with Poisson model: AIC = 662.1)

Predictor	Estimate	Std. error	Z value	p value
Intercept	− 3.682	2.643	− 1.393	0.164
Prevalence	27.063	8.905	3.039	0.002**
Sample size	0.232	0.093	2.485	0.013*
Prevalence^2^	− 18.229	8.694	− 2.097	0.036*

Significance codes: ** < 0.01, * < 0.1

## Discussion

In recent years, a great deal of effort has gone into studying the VAR2CSA protein, both in terms of its immune profile and its impact on clinical outcomes. However, while good data exists on levels of *var2csa* sequence polymorphism in several countries spanning four continents [15], to date no study has explored variation at this locus in samples obtained from DRC. More generally, understanding of the basic epidemiology and population genetics of this large and diverse country has been limited historically by a lack of good quality data. This study aimed to address this issue by quantifying *var2csa* variation in DRC and relating it back to basic epidemiological questions.

The results of this study demonstrate that *var2csa* is highly diverse within DRC, with 583 sequence variants identified among 812 children. High Tajima’s D and an excess of synonymous mutations suggest that this high diversity is being maintained by long-term balancing selection, as would be expected of a gene involved in antigenic variation, and as found in previous VAR2CSA studies [13–16]. However, there was no clear signal of phylogenetic structure to this diversity. Low bootstrap values in the neighbour joining tree indicate that different loci provide contradictory information about the position of sequences in the phylogeny, suggesting that recombination is acting to intertwine the phylogenetic branches and break down patterns across loci (see Additional file 2). In the spatial analysis, there was high heterozygosity within and between clusters but no signal of increasing genetic distance with geographic distance or other barriers to gene flow. Together these results indicate that the combined effects of rapid diversification through balancing selection, gene flow between spatial clusters, and recombination within the gene are acting to break down any clear signal of geographic population structure in this particular sub-region of *var2csa*. This contrasts with the result of Doritchamou et al. [16], who found clear signal of population structure in the N-terminal subregion just a few hundred bp upstream of the target region used here, in samples taken from Benin. However, when the Doritchamou et al. [16] sequences are truncated to the same target region used here, there is no clear signal of population structure (results not shown). Therefore, it appears that the extent of *var2csa* phylogenetic signal varies greatly even within narrow sub-regions of this highly diverse gene. It is not clear from this study what impact this diversity has on acquired immunity, however the excess of synonymous mutations suggests some role in diversification of the VAR2CSA antigen.

Results of general linear modelling demonstrate that *var2csa* diversity is directly related to epidemiological factors. The best-fitting model found that the allelic richness of a cluster tends to increase with prevalence, eventually plateauing out at high prevalence. Crucially, this analysis takes sampling effort into account—that is, prevalence is an important predictor of allelic richness even after accounting for the absolute number of infected children in the analysis. This result is in line with a wider body of evidence showing that genetic diversity tends to be higher in areas of intense transmission, perhaps due to increased opportunity for recombination [45–47]. In terms of vaccine design, this may indicate that a vaccine would be more likely to succeed in areas of low transmission where populations tend to be more clonal. The finding that common alleles in DRC also tend to be represented in other African countries (see Additional file 3) is also encouraging for vaccine development, as it indicates that some variants are common over large geographic scales, despite the general trend of strong local diversification.

One strength of this study is the use of samples derived from the DHS, which is both cross-sectional and nationally representative. This ensures that asymptomatic and low-density (i.e. sub-microscopic) infections are captured in the analysis, which may include a different subset of strains to those found in clinically ill individuals [48]. A caveat is that samples were extracted from children under 5 years of age, and not from infected pregnant women, and so the pool of strains sampled here may not perfectly reflect those implicated in clinical disease. The use of pooled samples also makes it possible to explore a wide geographic area, and to answer questions about spatial connectivity (Fig. 1). However, the use of pooled samples also limits analysis to making observations at the cluster level, and cannot reliably determine factors such as the complexity of infection of each individual within a cluster.

The results of this study highlight the extreme evolutionary pressures acting at the *var2csa* locus to promote antigenic variability in the DRC. This variability creates a substantial hurdle in the development of VAR2CSA-based vaccine, which is extenuated by the highly-connected population structure found within the DRC. A broad multivalent VAR2CSA vaccine candidate could thus benefit from targeting stable regions and common variants to address the substantial genetic diversity [49].

## Additional files

**Additional file 1.** Details of systematic literature search in PubMed and Web of Science, including full search terms and details of all studies identified.

**Additional file 2.** Neighbour-joining tree of samples from DRC, Benin and Senegal, produced in Figtree (http://tree.bio.ed.ac.uk/software/figtree/). Edges are coloured from red to blue according to their bootstrap percentage (black edges are terminal and so have no bootstrap value). Dotted lines leading away from the tree are coloured to indicate the origin of the sample.

**Additional file 3.** The number of clusters in which a variant was found plotted against percentage identity of top BLAST hit for that variant. Multiple variants have the same combination of BLAST percentage and cluster representation, so size of circles indicates the number of variants for a given combination.

**Additional file 4.** Regression of Nei’s (1972) genetic distance against great circle distance. The fitted relationship is non-significant (*ρ* = −0.04, *p* = 0.217).

**Additional file 5.** Best-fitting GLM compared to data. Black circles represent the median model prediction for each of the 115 clusters, and vertical bars represent the 95% predictive interval. Predictions are presented relative to the observed allelic richness, meaning the model is a good fit wherever the interval crosses the dashed zero line.
