# Supplementary figures and images for: Plasmodium falciparum genetic variation of var2csa in the Democratic Republic of the Congo
Source: Malar J. 2018 Jan 24;17:46. doi: 10.1186/s12936-018-2193-9 (PMC5782373; doi:10.1186/s12936-018-2193-9)

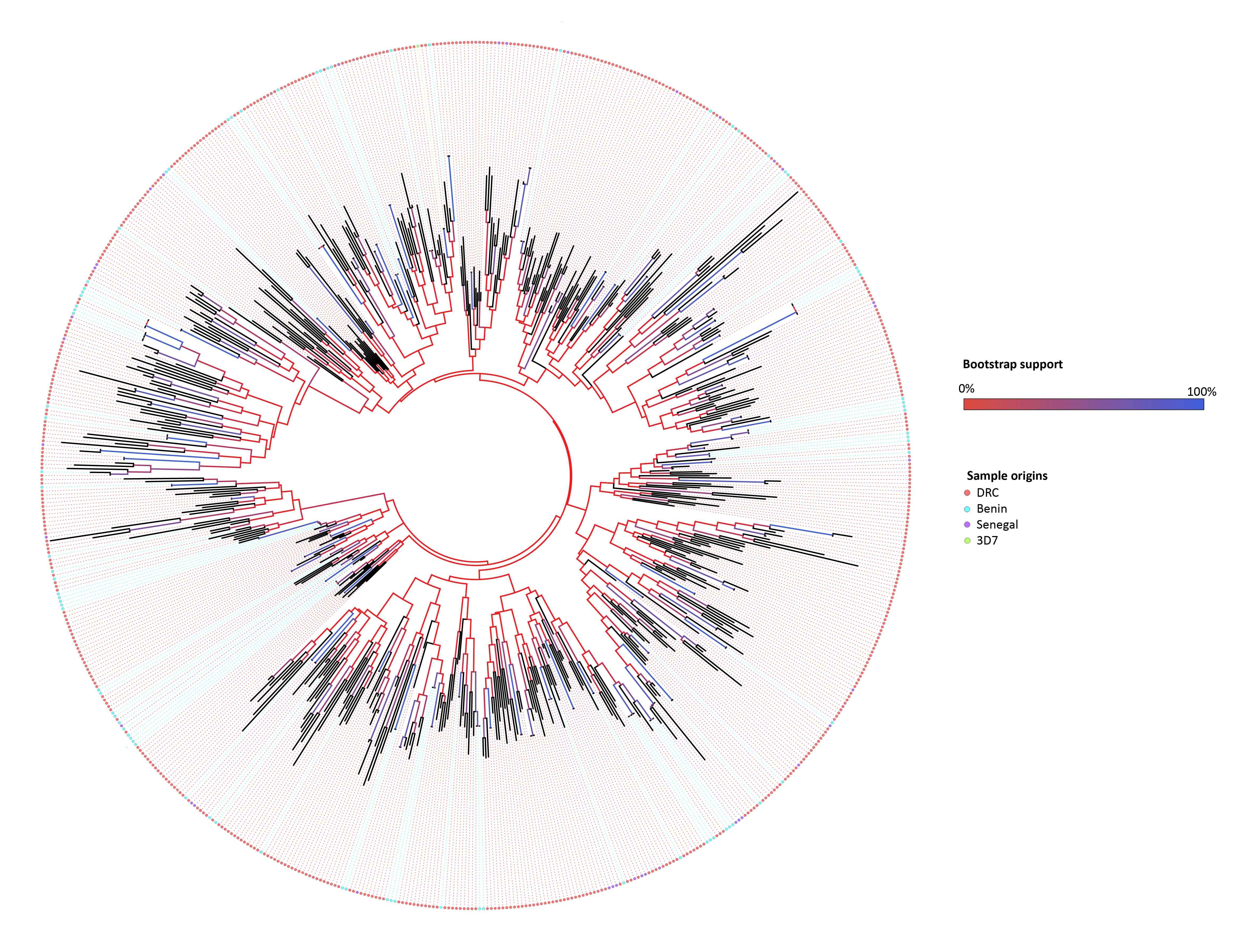

Supplement: Supplementary file 2 — Additional file 2. Neighbour-joining tree of samples from DRC, Benin and Senegal, produced in Figtree (http://tree.bio.ed.ac.uk/software/figtree/). Edges are coloured from red to blue according to their bootstrap percentage (black edges are terminal and so have no bootstrap value). Dotted lines leading away from the tree are coloured to indicate the origin of the sample. [file 12936_2018_2193_MOESM2_ESM.png]

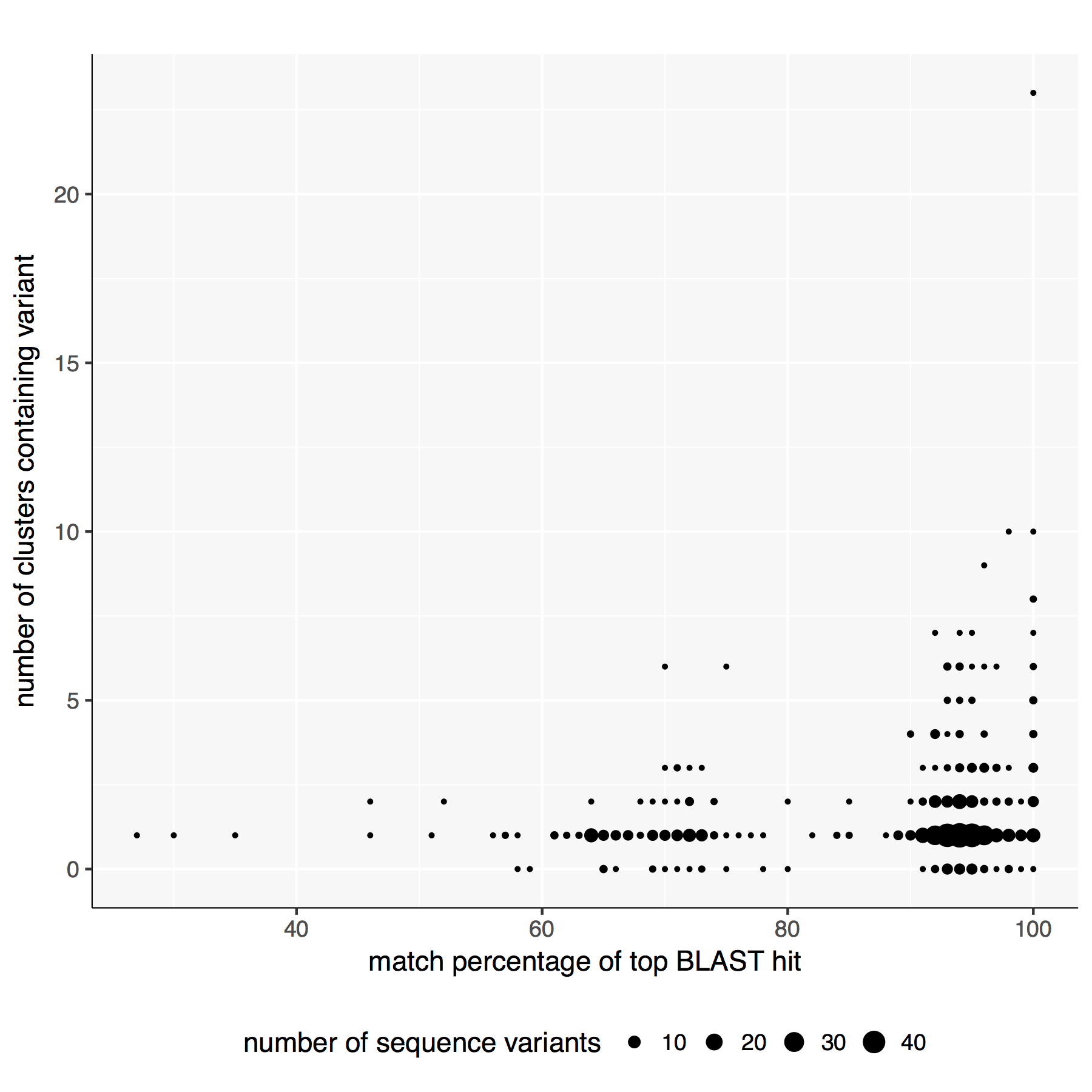

Supplement: Supplementary file 3 — Additional file 3. The number of clusters in which a variant was found plotted against percentage identity of top BLAST hit for that variant. Multiple variants have the same combination of BLAST percentage and cluster representation, so size of circles indicates the number of variants for a given combination. [file 12936_2018_2193_MOESM3_ESM.png]

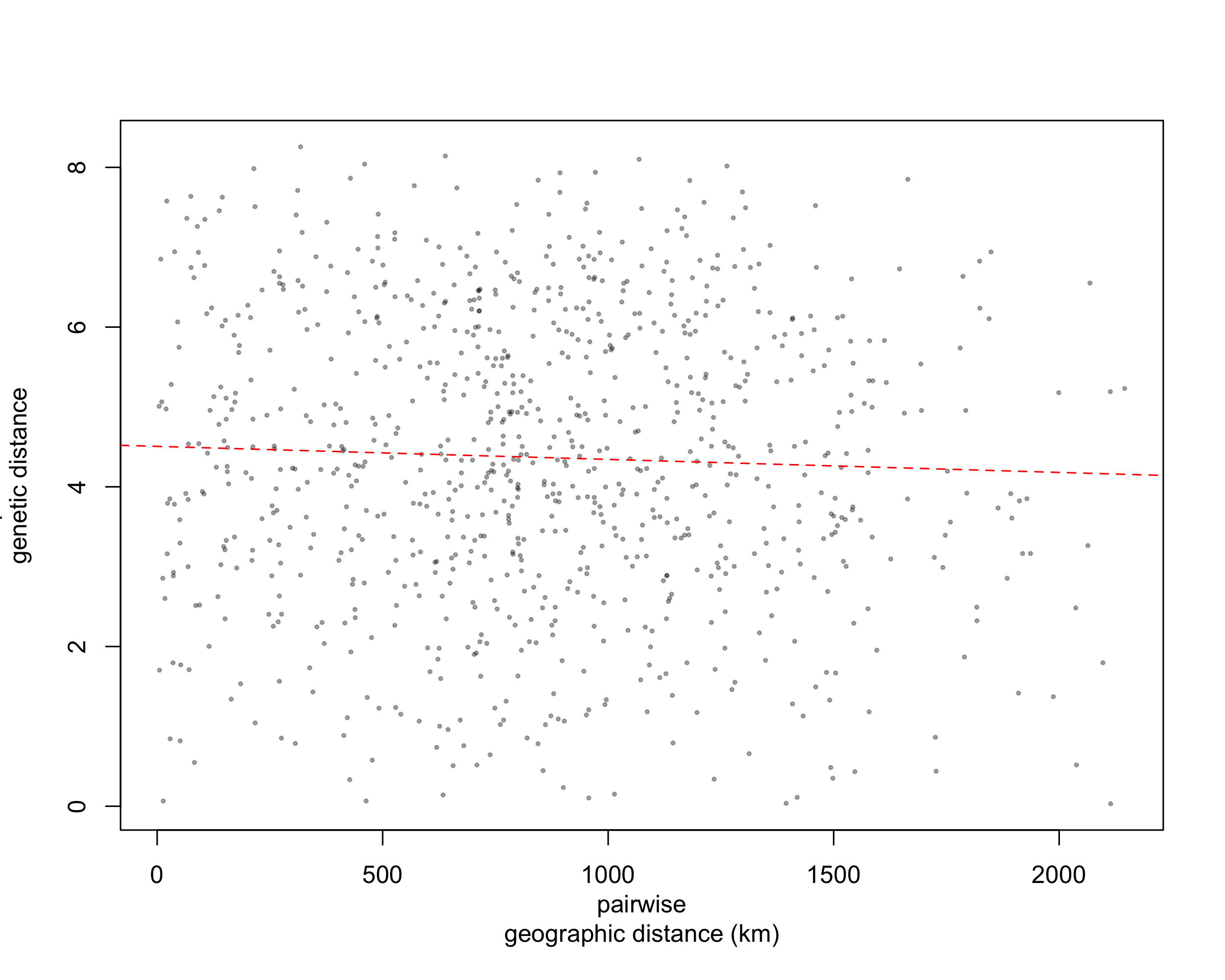

Supplement: Supplementary file 4 — Additional file 4. Regression of Nei’s (1972) genetic distance against great circle distance. The fitted relationship is non-significant (ρ = −0.04, p = 0.217). [file 12936_2018_2193_MOESM4_ESM.png]

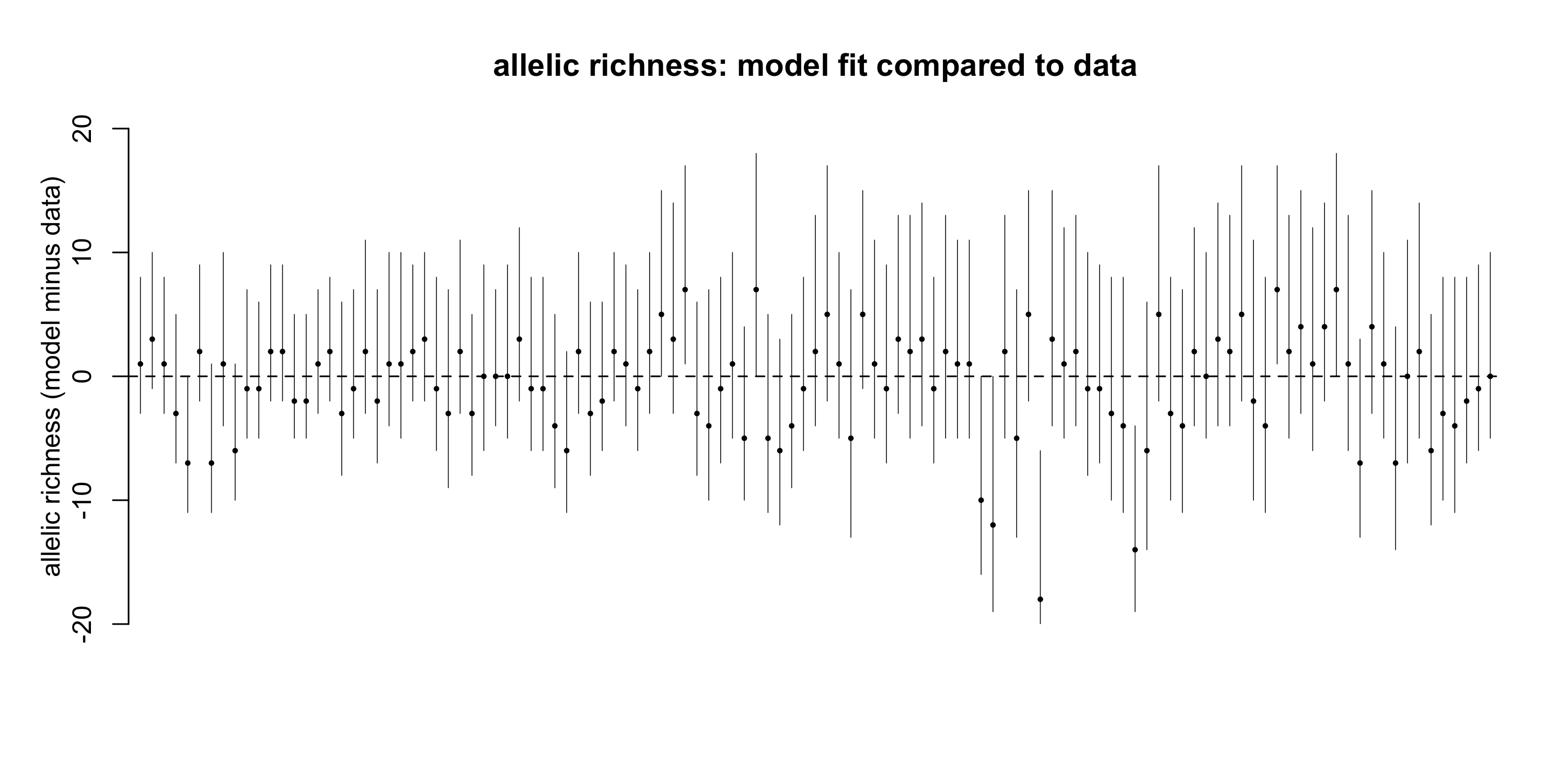

Supplement: Supplementary file 5 — Additional file 5. Best-fitting GLM compared to data. Black circles represent the median model prediction for each of the 115 clusters, and vertical bars represent the 95% predictive interval. Predictions are presented relative to the observed allelic richness, meaning the model is a good fit wherever the interval crosses the dashed zero line. [file 12936_2018_2193_MOESM5_ESM.png]
